# Impact of Digital Village Construction on Agricultural Carbon Emissions: Evidence from Mainland China

**DOI:** 10.3390/ijerph20054189

**Published:** 2023-02-26

**Authors:** Yue Zhang, Mengwei Feng, Zhengshuai Fang, Fujin Yi, Zhenzhen Liu

**Affiliations:** 1 School of Management, Hebei University, Baoding 071002, China; 2 School of Public Affairs, Zhejiang University, Hangzhou 310058, China; 3 College of Economics and Management, Nanjing Agricultural University, Nanjing 210095, China

**Keywords:** digital village, agricultural carbon emissions, nonagricultural employment, agricultural green development, mainland China

## Abstract

Reducing agricultural carbon emissions is required to reach the goal of carbon neutrality and mitigate the effects of climate change. With the advent of the digital economy, we aimed to determine if digital village construction can achieve carbon reduction in agriculture. As such, in this study, we used balanced panel data for 30 provinces in China from 2011 to 2020 to conduct an empirical analysis based on measuring the digital village construction level in each province. We found the following: Firstly, digital village construction is conducive to reducing the carbon emitted from agriculture, and the results of further tests showed that the carbon reduction effect of digital villages is mainly based on the reduction in carbon emissions from chemical fertilisers and pesticides. Secondly, the digital village construction has a stronger inhibiting effect on agricultural carbon emissions in major grain-producing areas than in non-major grain-producing areas. The level of rural human capital is the limiting condition for digital village construction to enable green agricultural development; in areas with higher levels of human capital, digital village construction has a significant inhibiting effect on agricultural carbon emissions. The above conclusions are valuable for the future promotion of digital village construction and the design of a green development model for agriculture.

## 1. Introduction

Recently, Chinese agriculture has rapidly advanced. Grain production has increased from 304.75 million tonnes in 1978 to 669.49 million tonnes in 2020 (Data source: National Bureau of Statistics of China). At the same time, the growth rate of grain consumption per capita has reached 61.62%. However, Chinese agricultural production has long followed the high input–high output model; the use of pesticides, chemical fertilisers, and agricultural films has become an important means through which peasants increase production and income [1]. High agricultural inputs result in large agricultural carbon emissions, and the greenhouse gases produced by global food systems accounted for 34% of the total global greenhouse gas emissions caused by humans in 2015 [2]. The glacial melting and extreme weather caused by the greenhouse effect, which is mainly caused by massive carbon emissions, are already seriously threatening human production and life. In 2020, at the 75th session of the UN General Assembly, China committed to achieving the carbon peak by 2030 and carbon neutrality by 2060. The reduction in agricultural carbon emissions is not only a response to people’s desire for a beautiful environment but is necessary for reaching these targets in China.

In order to reach the targets, it is necessary to enhance the endogenous power of carbon reduction in agriculture from the following two perspectives. Firstly, peasants’ perceptions of the necessity and benefits of green production profoundly influence their production behaviour [3]. The effective transmission of environmental and market information will encourage peasants to engage in green produce and achieve carbon reduction in agriculture. Secondly, technology is an endogenous driver of economic development and a source of economic growth. Technological advances have become the engine of the green economy, and technology is an important factor in reducing carbon emissions in agriculture [4].

Since the commercialisation of the Internet in the 1990s, the mobile Internet, big data, and other technologies have created new momentum in terms of economic development, especially rural development. With the development of digital technology, new elements represented by information and technology will penetrate into all aspects of agricultural production, peasants’ lives, and rural development, thus producing an overall improvement in the quality, efficiency, and dynamics of village development [5]. Therefore, promoting the construction of digital villages has become a new direction for future rural development. In this study, we defined digital village construction as an economic activity based on the use of information and technology elements in agricultural production and rural economic development, based on constantly upgraded digital facilities [6,7]. Digital village construction has become a new method to hasten the transformation of agricultural and rural development methods. Through the benefits produced by information and technology for agricultural production in digital villages, the entire agricultural industry chain and rural society will be better informed, which will promote the upgrading of agricultural production methods and peasants’ production skills. Therefore, digital village construction is an effective path to stimulate the speed of rural development, thus promoting rural revitalisation and sustainable development and playing an endogenous driving role in achieving carbon emission reduction in agriculture.

When studying digital villages, most researchers used qualitative analysis to explore their connotations, mechanisms, dilemmas, and future development direction [7,8,9]. In the past year, quantitative research on digital villages has been gradually enriched. However, in general, more studies on the economic or social effects of digital village construction are still needed. In the field of resources and environment, Hao et al. [10] analysed the impact of digital village construction on overall rural carbon emissions, including living, production, and electricity, but did not focus on agricultural production carbon emissions alone, which led to a difference in their analysis of its underlying mechanism in this paper. Rao et al. [11] focused on the relationship between rural broadband adoption and agricultural carbon emission reduction efficiency. However, rural broadband adoption is only one dimension in the construction of digital villages, which cannot fully reflect the whole picture of digital village construction. Huang et al. [12] used micro-individual data to test the impact of digital technology on low-carbon production behaviour of farm households. Can digital village construction promote agricultural carbon reduction? There is still a lack of detailed mechanism analysis and data testing concerning this question.

As such, we focused on the impact of digital village construction on agricultural carbon emissions. The aim was to provide theoretical and empirical evidence to support the green development of agriculture through the digital transformation of villages. The innovations of this study are as follows: On the one hand, based on the real demand of green agricultural development, this paper uses provincial-level data in mainland China to form an index system for evaluating the level of digital village construction and, on this basis, focuses on the effect of digital village construction on the total carbon emissions of agricultural production, which is an effective supplement to the current research on digital village construction. On the other hand, this paper further analyses the differential impact of digital village construction on different types of agricultural carbon emissions and tests the heterogeneity of different regions. From this perspective, this paper clarifies the inner mechanism of the impact of digital village construction on agricultural carbon emissions.

## 2. Conceptual Framework

Since the introduction of digital villages, digital technology has been rapidly penetrating the countryside. Digital technologies are creating new opportunities for agriculture, rural areas, and peasants, and promoting the green transformation of agriculture. Specifically, the digital village construction impacts agricultural carbon emissions through its technological and information-driven effects.

### 2.1. Technological Innovation Effect of Digital Village Construction

Digital village construction promotes agricultural carbon reduction through the effect of technological innovation [11]. Improving rural digital infrastructure and promoting the spread of digital applications are the basic aims of digital village construction [7]. Relying on the compatible, intensive, and extended features of digital technology, digital village construction considerably reduces the cost of agricultural technology innovation and promotes the intelligent and informative transformation of agricultural production links [5]. The lack of access to knowledge is preventing agricultural producers from adopting green production techniques. In the digital age, knowledge is noncompetitive. Through the construction of digital villages, agricultural producers can quickly acquire knowledge about green agricultural production through mobile communication devices and the Internet, which reduces the cost of information searching and thus breaks through this limit faced by agricultural producers in adopting new technologies. Based on the popularisation of basic digital technologies, such as the Internet and the Internet of Things, and the improvement in rural digital infrastructure, the transformation and upgrading of the agricultural production chain, including operations and monitoring, will be achieved, thus promoting the green transformation of agricultural production [13]. For example, data mining technology can be used to effectively integrate data on seedlings, cultivation, pests, and diseases, and to implement dynamic monitoring. Combined with remote sensing and Internet of Things technologies, data mining can be used to achieve precision irrigation, soil formula fertilisation, precise pest control, and waste recycling, thus promoting the reduction in and reuse of chemical inputs and reducing carbon emissions in agriculture [14].

### 2.2. Information-Driven Effect of Digital Village Construction

Information is an important factor driving production [15]. Peasants’ access to information in the past was mainly through books, newspapers, radio, television, and interpersonal communication. With the penetration of digital technology, new media represented by the Internet and new businesses such as e-commerce have gradually become effective avenues through which peasants can obtain information. Digital village construction provides a wealth of information resources for the development of agriculture, rural areas and peasants. Technologies such as big data and cloud computing enable the efficient collection and integration of information; Internet technologies enable the rapid sharing of information; terminals such as mobile communication devices enable the multidimensional display of information. Information visualisation theory suggests that by directly transforming information that is difficult to display into visual representations such as graphics and video, the reception of information by individuals can be markedly enhanced [16]. The production and management decisions of agricultural producers have long been characterised by a certain short-sightedness with the excessive pursuit of economic profit, which has led to a lack of willingness to reduce chemical inputs and adopt green production techniques [17]. Constructing digital villages will change this through an information-driven effect, as follows.

Firstly, the information-driven effect of digital village construction enables peasants to better connect to the market and understand current market demand. Over time, consumer demand for agricultural products has become more diversified and their willingness to buy and pay a premium for green produce is gradually increasing [18]. Promoting the development of new businesses in rural areas, such as digital finance and e-commerce with Internet technology as the core, is a requirement for digital village construction. Digital finance and e-commerce both produce a strong information effect. They make it easier for agricultural producers to access external information related to production and operation with the help of the Internet platform and to realize the direct connection between consumers and producers [19,20]. Therefore, the construction of a digital village breaks down the information barriers between agricultural producers and consumers and enables agricultural producers to grasp consumers’ desires for green and healthy food [21]. In China, most agricultural producers tend to emphasise their control of chemical inputs such as pesticides as a way to prove that their products are green and healthy. For example, in the pest resistance process, peasants will use trapping mechanisms or solar heat for physical control, or innovative biological control measures. When using pesticides, the number and concentration of doses must be controlled to meet the standards of green and healthy food. In the fertilisation process, peasants will choose farmyard manure instead of chemical fertilisers. The above initiatives are effective in reducing agricultural carbon emissions while achieving green production. Therefore, the digital village construction can reduce agricultural carbon emissions by stimulating peasants’ willingness to adopt green production through information effects, which, in turn, can reduce chemical inputs or increase chemical use efficiency.

Secondly, the information-driven effect of digital village construction keeps peasants abreast of policy and environmental information. The widening of information access allows peasants to learn about agricultural and rural development policies without having to leave their homes while educating themselves on the seriousness of environmental pollution problems and the importance of environmental management through diversified information access channels. This, in turn, raises peasants’ awareness of environmental protection and motivates them to adopt green production techniques and reduce chemical inputs, thereby influencing agricultural carbon emissions.

In summary, we propose the following hypothesis:

**Hypothesis** **1** **(H1):** *Digital village construction promotes lower agricultural carbon emissions.*

## 3. Data and Variables

### 3.1. Description of Data

In this study, we selected balanced panel data consisting of 30 provincial administrative regions across China from 2011 to 2020 as the sample [22]. The data sources for each indicator within the digital village construction level evaluation system are described in the following section. Some values were missing for rural computer ownership per 100 households and rural mobile phone ownership per 100 households for 2013 and 2014. Therefore, we extrapolated the corresponding data from the average growth rates of other years. We obtained data on the variables in the empirical analysis from the National Bureau of Statistics, the China Rural Statistical Yearbook, the China Tertiary Industry Statistical Yearbook, and the China Labour and Employment Statistical Yearbook.

### 3.2. Definition of Variables

(1) Explained variable: Agricultural carbon emissions. The main sources of carbon emissions from agriculture include chemical fertilisers, pesticides, agricultural films, agricultural diesel, irrigation, and tillage [23]. The formula for calculating agricultural carbon emissions is as follows:

(1) $$CE=\sum T_{i}\times \rho_{i}$$

where *CE* represents agricultural carbon emissions, $T_{i}$ is the consumption of carbon emission source *i*, and $\rho_{i}$ is the coefficient of carbon emission source *i*. Table 1 shows the sources of agricultural carbon emissions, coefficients, and references. The carbon emission coefficient of chemical fertiliser, pesticide, agricultural film, agricultural diesel consumed by agricultural machinery, agricultural irrigation, and agricultural tillage are 0.8956 kg/kg, 4.9341 kg/kg, 5.1800 kg/kg, 0.5927 kg/kg, 19.8575 kg/hm^2^ and 312.6 kg/km^2^, respectively.

(2) Explanatory variable: Level of digital village construction. Digital village construction is a multifactorial, multidimensional, and comprehensive task that is difficult to measure using a single indicator. Therefore, we selected indicators from multiple perspectives to form a digital village construction evaluation index system.

In this study, considering prior studies [7,10,26], we selected nine indicators from the three dimensions of digital infrastructure, industry digitisation, and digital services to constitute an index system for evaluating the level of digital village construction (Table 2). The reasons are as follows: (a) Digital infrastructure is the foundation of digital village construction. The Internet, computers, and mobile devices are the basis for the digital production and enjoyment of life in rural areas. Therefore, accessibility to the Internet and digital devices is an important indicator in evaluating the level of digital village construction. (b) Industrial development is the key to rural economic development, and digitisation of rural traditional industries is the core of digital village construction. The industry digitisation dimension selects indicators from three perspectives: agricultural production, sales, and financial industry. National modern agricultural demonstration zones and industrial parks are built according to modern agricultural standards, which are highly compatible with the construction of digital villages and can reflect the digital level of agricultural production. The construction of Taobao Villages reflects the level of rural e-commerce development and is a valid metric for the digitisation of sales. The digital finance digitisation index reflects the level of convenience and cost of digital finance and is a reflection of the digitisation of the financial industry [27]. (c) The goal of digital village construction is to effectively transform peasants’ lifestyles. The digital financial coverage breadth index reflects the level of peasants’ acceptance of the new financial industry and is a reflection of the changes in the way peasants access financial services. E-commerce services reflect changes in peasants’ purchasing and selling behaviours. The level of mobile payment reflects the digital upgrading of peasants’ means of payment.

In the calculation of the indicators, we normalised each indicator with reference to Melnychuk et al. [28]. For example, fixed digital device accessibility used computer ownership per 100 households, and e-commerce services used the number of weekly deliveries. However, some indicators (e.g., digital financial services) are derived from the Peking University Digital Inclusive Finance Index, which has been normalised.

Next, we used factor analysis to synthesise the indicators of the level of digital village construction based on the digital village construction level evaluation index system described in Table 2. The steps we used to calculate the level of digital village construction using factor analysis were as follows:

(a) As different indicators have different scales, we firstly adopted the mean value method for standardisation. The averaging method was calculated using the following formula:

(2) $$y_{ij}=x_{ij}/{\bar{x}}_{j}$$

where $x_{ij}$ is the raw data of indicator *j* for region i, and ${\overset{-}{x}}_{j}$ is the mean value of indicator *j*.

(b) We applied the KMO (Kaiser-Meyer-Olkin) and Bartlett sphericity tests to determine the existence of a certain correlation between the indicators. The KMO statistic was calculated using the following formula:

(3) $$KMO=\frac{\sum \sum_{i\neq j}r_{ij}^{2}}{\sum \sum_{i\neq j}r_{ij}^{2}+\sum \sum_{i\neq j}p_{ij}^{2}}$$

where $r_{ij}$ is the simple correlation coefficient between indicator *i* and indicator *j*, and $p_{ij}$ is the partial correlation coefficient between indicator *i* and indicator *j* with the remaining indicators controlled. The larger the KMO value, the stronger the correlation between indicators and the more suitable it is for factor analysis. Typically, we require a KMO value between 0.5 and 1.0. The statistic of Bartlett’s sphericity test is calculated based on the determinant of the correlation coefficient matrix and approximately follows a chi-square distribution. If this statistic is large and the corresponding probability value is less than the given significance level, it is considered suitable for factor analysis. The results of the KMO and Bartlett sphericity tests are presented in Table 3.

The test results showed that the KMO value was 0.833 and the probability of Bartlett’s sphericity test was 0.00. These results indicated that the indicators we selected were suitable for factor analysis.

(c) Based on the principle that the eigenvalue is larger than 1, the calculation results showed that the cumulative variance contribution rate of the extracted principal components was 73.566%, indicating that the extracted factors have good explanatory power for the construction level of digital villages (Table 4).

(d) We orthogonally transformed the extracted factors using the maximum variance method. After that, we obtained the component score coefficient matrix, and calculated the component score for each province accordingly.

(e) We used the percentage of the variance contribution of each component to the cumulative variance as the weight of each component. The weights of the two components were 0.5875 and 0.4125. Then, we calculated the level of digital village construction.

(3) Control variables: With reference to prior studies [27,29,30], we selected the rural economic level, agroindustrial structure, rural human capital, regional innovation level, and urbanisation rate as control variables. Firstly, we used the level of peasants’ net income to measure the rural economic level [30]. Secondly, for the agroindustrial structure, we used the ratio of total agricultural output to total agricultural, forestry, animal husbandry, and fishery output as a measure [29]. Thirdly, the level of education is an important reflection of human capital [31]. We measured the level of rural human capital using the weighted average education level of rural residents, based on Barro and Lee [32] with reference to the educational situation and data availability in China, calculated as follows:

(4) $$RHC_{i,t}=\sum_{m=1}^{k}r_{m,i,t}H_{m,i,t}$$

where $RHC_{i,t}$ is the rural human capital in province *i* at time *t*; *m* = 1–5 represent the five education levels, i.e., uneducated, primary, junior secondary, senior secondary, and tertiary and above, respectively; r represents the ratio of the rural labour force with a particular education level in the total labour force; and H represents the number of years of education at a particular education level, where H (uneducated) = 0, H (primary) = 6, H (junior secondary) = 9, H (senior secondary) = 12, and H (tertiary and above) = 15. Fourthly, considering that the number of patents granted better reflects the real innovation capability than the number of patent applications, this paper used the logarithm of patents granted per 10,000 population to measure the regional innovation level. Fifthly, referring to prior studies [27,30], we used the ratio of urban population to total population to measure the urbanisation rate. The definitions of all the variables selected in this paper are shown in Table 5.

## 4. Research Findings and Analysis

### 4.1. Descriptive Statistics

**Figure 1:**
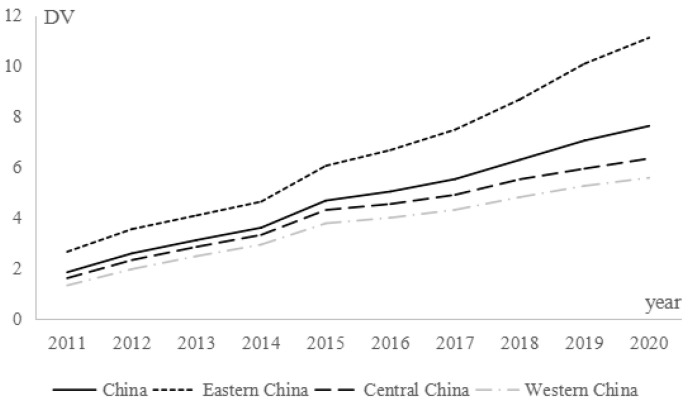
Trends in the level of digital development in rural areas.

**Figure 2:**
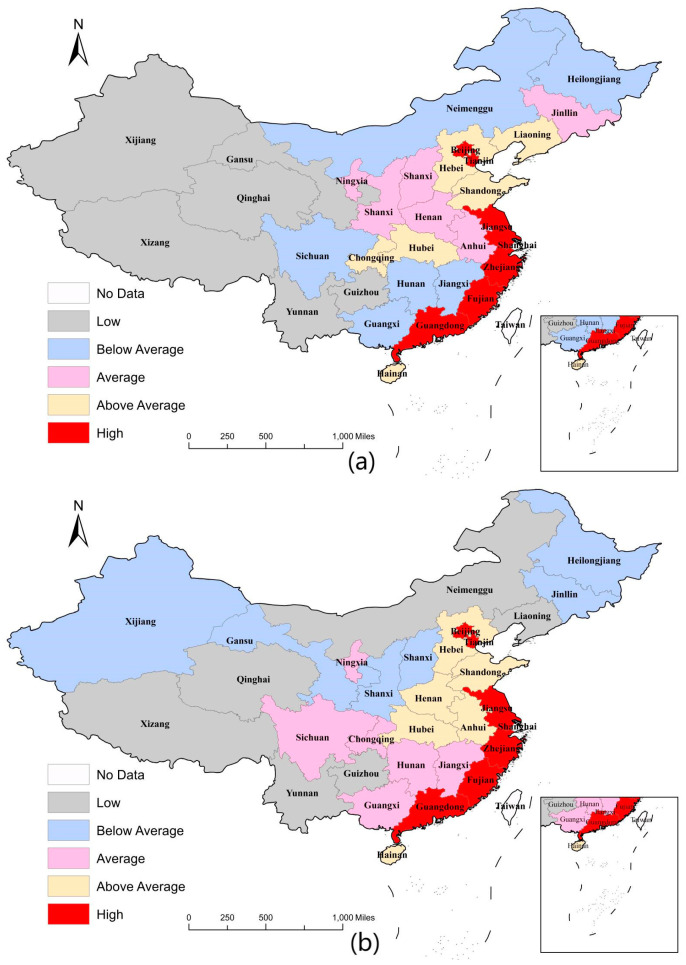
Spatial distribution of digital village construction level in 2011 (**a**) and 2020 (**b**).

The results of the descriptive statistics for each variable are shown in Table 5, where the mean value of digital village construction is 4.724. Figure 1 shows the trend in the level of digital village construction, from which we found that the digital village construction index basically showed an increasing trend. From 2011 to 2020, the growth rate of China’s digital village construction level was 317.21%, with an average annual growth rate of 17.20%. By region, the level of digital village construction in the east, middle, and west maintained an upward trend, with average annual growth rates of 17.33%, 16.57%, and 17.49%, respectively, indicating that all regions in China have achieved certain results in the construction of digital villages. At the same time, there are obvious regional differences in the construction of digital villages. Figure 1 shows that the digital village construction level was higher in the eastern region than the national average in all years. The east was ahead of the central and western regions. Figure 2 shows that, no matter whether in 2011 or 2020, the areas with a high level of digital village construction are on the east coast, including Beijing, Tianjin, Jiangsu, Zhejiang, Fujian, and Guangdong. The second-tier provinces are mainly in the eastern and central regions, such as Hebei, Hubei, and Shandong. The construction level of digital villages in western China was generally low. Overall, the construction of digital villages in China presents the characteristics of “strong in the east, transitional in the middle and weak in the west”. The reasons for this feature are as follows: The eastern region has obvious resource advantages and policy dividends, and digital technology here sprouted early, developed rapidly, and penetrated into rural areas quickly, thus taking the lead in the construction of China’s digital villages. However, capital, technology, talent, and other resources in the western region are insufficient, so the foundation for digital village construction is lacking.

The mean value of agricultural carbon emissions was 28.809, and had a standard deviation of 19.686, indicating a large variation in these carbon emissions between provinces. The average rural economic level was 9.332 with a standard deviation of 0.381. The average value of the agricultural industry structure was 52.731, while the average value of rural human capital was 7.643, indicating that the overall level of education in rural areas needs to be increased. The average value of the regional innovation level was 2.014, and that of the urbanisation rate was 56.374. Before regression, we firstly conducted a multicollinearity test. The results of the VIF test showed that the maximum VIF was 6.960, and the correlation coefficients between the explanatory variables and the control variables were all less than 0.8, thus proving that multicollinearity was not a problem.

### 4.2. Impact of Digital Village Construction on Agricultural Carbon Emissions

Next, this paper examined the impact of digital village construction on agricultural carbon emissions. For panel data, mixed regression, fixed effects models, or random effects models are usually used. In this study, we firstly conducted an F test, which showed that the F statistic was 666.59 and the corresponding probability value was 0. The F test result showed that the fixed effect model was better than the mixed regression. After that, we conducted Hausman tests, and the test result showed that the $\chi^{2}$ statistic was 13.56, corresponding to a probability of 0.060. The Hausman test result showed that the random effects model was better than the fixed effects model. To ensure robust results in this study, we first ran regressions without the inclusion of control variables, after which we added control variables. Next, the regression results were presented for the inclusion of annual control variables versus those without. By adding annual control variables, the impact of policy changes (e.g., chemical reduction policies) and inflation was effectively controlled. The results are shown in Table 6.

**Figure 3:**
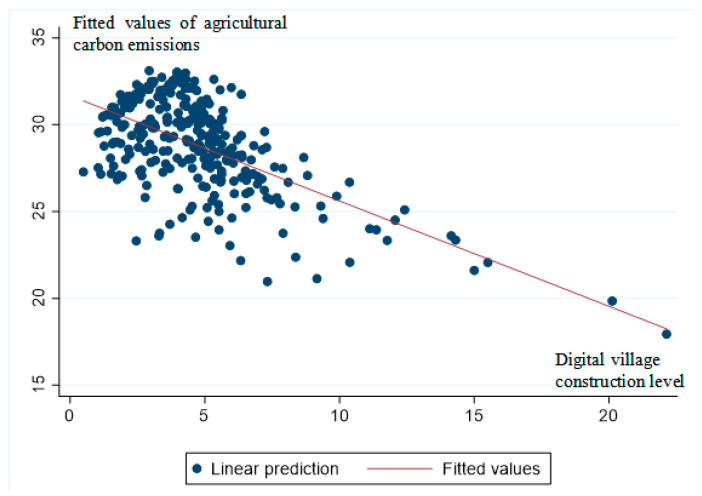
Fitted line graph.

In Table 6, the results of the Wald test indicate that the explanatory power of the model is strong (the random effects model uses maximum likelihood estimation (MLE), so the Wald test is used to judge the significance of the model. When the Wald statistic is greater than the critical value and the corresponding probability value is lower than the given significance level, the model is considered to have a strong explanatory power). Columns (1) and (2) show the regression results for the uncontrolled years. Based on the results of data regression, we found that the level of digital village construction was significant at the 1% level with negative values, regardless of whether control variables were included, which indicated that digital village construction reduced agricultural carbon emissions. Columns (3) and (4) provide the regression results with the addition of annual control variables. Column (3) is significant at the 5% level and has a negative value for the level of digital village construction when no control variables are included. Column (4) is significant at the 1% level of significance and has a negative value for the level of digital village construction after the inclusion of control variables. For every one-unit increase in the level of digital village construction, agricultural carbon emissions are reduced by 0.291 million tonnes, which proves that digital village construction has promoted the reduction of agricultural carbon emissions. The construction of digital villages stimulated the subjective will of agricultural producers to engage in green practices through access to information dividends. Digital village construction simultaneously enabled the rapid dissemination of knowledge through the Internet and mobile device terminals and promoted the mastery of the latest production techniques by agricultural producers. Additionally, digital village construction enabled technological advances to be used to promote technological innovation in green agricultural production. Figure 3 shows the fitted line graph after regression, indicating that digital village construction does have a negative effect on agricultural carbon emissions. Therefore, H1 was supported.

To further investigate the mechanism through which digital village construction impacted agricultural carbon emissions, we then examined the effect of digital village construction on four different types of carbon sources: chemical fertilisers, pesticides, agricultural films, and machinery fuel. We did not examine the impact of digital village construction on carbon emissions from tillage and irrigation, mainly for the following reasons: In terms of data construction, carbon emissions from tillage and irrigation mainly depend on the area sown and the area effectively irrigated, and these figures do not considerably change due to the information-driven and technological innovation effects of digital village construction. From this perspective, examining the impact of the construction of digital villages on the carbon emissions of agricultural machinery fuel and chemical inputs more strongly supports the validity of the previous mechanism. The specific regression results are shown in Table 7, which show that digital village construction mainly reduced carbon emissions from chemical fertilisers and pesticides, although the effect on carbon emissions from agricultural films and machinery fuel was negative, albeit not significant. These results suggest that digital village construction promoted reductions in chemical inputs and agricultural carbon emissions through technological innovation and information-driven effects.

### 4.3. Robustness Tests

(1) Substitution of explanatory variables

The results of factor analysis can reflect most of the information in the original indicator with fewer mutually independent factors, resulting in an indicator dimensionality reduction. In addition, the entropy method of determining indicator weights to produce a comprehensive indicator was a common practice in prior studies [10,33]. As such, we determined the weight of each indicator (Table 2) according to the entropy value method. The steps were as follows:

(a) We standardised the data of the nine indicators in Table 2, and the calculation formula is as follows:

(5) $${x^{\prime }}_{i,j}=\frac{x_{i,j}-x_{j(\min)}}{x_{j(\max)}-x_{j(\min)}}$$

where $x_{i,j}^{\prime }$ is the value of province *i* after standardisation of the *j* index, $x_{i,j}$ is the original value of indicator *j* for province *i*, $x_{j(\min)}$ is the minimum value of indicator *j* for all provinces in the sample period, and $x_{j(\max)}$ is the maximum value of indicator *j* for all provinces in the sample period.

(b) We calculated the proportion of standardised data of the j index in province *i* ($y_{i,j}$). The formula is as follows:

(6) $$y_{i,j}=\frac{x_{i,j}^{\prime }}{\sum_{i=1}^{n}x_{i,j}^{\prime }}$$

(c) We calculated the information entropy of the j index ($\varphi_{j}$). The formula is as follows:

(7) $$\varphi_{j}=-c\sum_{i=1}^{n}y_{i,j}\ln y_{i,j}$$

In the above formula, c=1ln(n).

(d) We calculated the weight of the j index with the following formula:

(8) $$w_{j}=\frac{g_{j}}{\sum_{j=1}^{9}g_{j}}$$

In the above formula, $g_{j}=1-\varphi_{j}$.

(e) We generated the digital village construction level 2 (DV2) according to Formula (9):

(9) $$DV2_{i}=\sum_{j=1}^{9}w_{j}x_{i,j}^{\prime }$$

Afterward, we again regressed the digital village construction level 2 calculated from Formula (9) to ensure the robustness of the results. The regression results are shown in column (1) in Table 8, indicating that the previous conclusions were robust.

(2) Fixed effects model

In the previous section, we demonstrated through the Hausman test that a random effects model regression should be used, but a fixed effects model can help with alleviating the endogeneity problems caused by omitted variables. As such, we also used a fixed effects model for regression to ensure the robustness of the results. As can be seen from the results in column (2) in Table 8, the previous conclusions still held based on the regression using the fixed effects model.

(3) Considering time lag effects

The construction of digital villages may take time to affect agricultural carbon emissions, so we again regressed agricultural carbon emissions as the explanatory variable in the period t + 1. The results are shown in column (3) in Table 8; the coefficient of the digital village construction level was still significantly negative, and the previous conclusion was found to be robust.

(4) Instrumental variables method

We mainly focused on the impact of digital village construction on agricultural carbon emissions; however, a reciprocal causal relationship may exist between them. Despite choosing as many control variables as possible in this study, the endogeneity problems arising from omitted variables cannot be ignored. Endogeneity issues can lead to inconsistent estimation results. As such, we used the instrumental variables approach to run the regression again.

In this study, we selected instrumental variables from two perspectives: Firstly, referencing Wei [34], we chose the 1984 per capita fixed telephone ownership as an instrumental variable to indicate the level of digital village construction. The internal logic of this instrumental variable is that the Internet was initially accessed by dial-up telephone, so it could be assumed that the application of Internet and digital technology began with fixed telephones; therefore, fixed telephone ownership per capita was closely related to the construction of digital villages, satisfying the correlation requirement of the instrumental variable. Fixed telephone ownership per capita in 1984 had essentially no impact on current economic development, so the instrumental variable satisfied the exogeneity requirement. However, as we used panel data, and the 1984 per capita fixed telephone ownership data are cross-sectional, we adopted the 1984 per capita fixed telephone ownership Internet penetration lagged by one period as the first instrumental variable for determining the level of digital village construction. Secondly, the level of digital village construction was closely related to the level of attention paid by local governments to this work and related policy pressure. Following this idea, we used the text mining method. We used “digital agriculture”, “smart agriculture”, “big data in agriculture”, “Internet finance”, “digital finance”, “financial technology”, and “digital economy” as keywords in this study, and we used the logarithm of the number of the above keywords + 1 in each annual government work report as the second instrumental variable. Theoretically, the above instrumental variables satisfy the correlation requirement. In terms of exogeneity, the extent to which local governments pay attention to the digital village construction must act on the real economy through practical actions (improving the construction of digital villages), so the instrumental variables in this study satisfied the exogeneity requirement.

The results of the regression using the instrumental variables method are shown in Table 9. For the choice of estimation methods, both Generalized Method of Moments (GMM) and Two Stage Least Square (2SLS) were used in this paper to ensure robustness. When using the 2SLS method, this paper was still based on the random effects model. In order to verify the validity of the instrumental variables, we carried out an underidentification test, a weak identification test, and an overidentification test. According to the regression results of the first stage (columns (1) and (3) in Table 9), in the underidentification test and weak identification test, the instrumental variables were correlated with endogenous explanatory variables. In the overidentification test, the Hansen J statistic was 0.130, corresponding to a probability of 0.719. As such, the original hypothesis was accepted, indicating that the instrumental variables satisfied the exogeneity requirement. The above results prove that the instrumental variables selected in this paper are reasonable. The regression results showed that the coefficient for the level of digital village construction was negative at the 1% significance level (columns (2) and (4) in Table 9), thus proving that the previous conclusions still held true after controlling for endogeneity.

### 4.4. Heterogeneity Analysis of Impact of Digital Village Construction on Agricultural Carbon Emissions

(1) Impact of digital village construction on agricultural carbon emissions in different regions

The factor endowments and development bases of agricultural development in different regions were substantially different, which could have affected the effects of digital village construction on agricultural carbon emissions. In this study, we grouped the regressions as major and non-major grain-producing areas, and the results are shown in columns (1) and (2) in Table 10. From the results, we found that digital village construction had a significant negative impact on the agricultural carbon emissions in both regions. Comparing the values of the coefficients, we found that digital village construction had a stronger inhibiting effect on agricultural carbon emissions in the major grain-producing areas. In major grain-producing areas, for every one-unit increase in the level of digital village construction, agricultural carbon emissions were reduced by 0.731 million tonnes. In non-major grain-producing areas, for every one-unit increase in the level of digital village construction, agricultural carbon emissions were reduced by 0.266 million tonnes. Compared with non-major grain-producing areas, major grain-producing areas have stronger agricultural resource endowments and policy dividends, especially in terms of arable land protection and financial subsidies, which are better than those in non-food-producing areas. With the advent of the digital economy, major grain-producing areas have been actively exploring methods to achieve digital transformation in agriculture. Therefore, the impact of digital village construction on agricultural carbon emissions in the major grain-producing areas is stronger. The major grain-producing areas contribute more than 70% of China’s grain production, and geographically, most of China’s major grain-producing areas are located in key ecological zones, so the major grain-producing areas occupy an important position in China’s agricultural production and ecological protection. Digital village construction played a stronger role in curbing agricultural carbon emissions in the major grain-producing areas, which is in line with both the theoretical expectations and the realistic need to develop green agriculture in China.

(2) Heterogeneity in level of human capital

With the development of the digital economy, the inequality in opportunity produced by new technological changes has become a growing concern for academics. The digital divide, which is wide between regions, between rural and urban areas, and between different groups of people, affects the empowering effect of digital technology on different regions and different groups of people. Digital village construction has bridged the primary divide between urban and rural areas, represented by the level of access to digital technology. However, the secondary digital divide, represented by digital capabilities, is creating new stratification characteristics in the dimensions of economic gains, welfare levels, and political participation, which also limit the benefits of digital village construction. The level of human capital reflects the learning ability of peasants, which further influences their ability to apply digital technology and information data in the digital economy. Therefore, the inhibiting effect of digital village construction on agricultural carbon emissions may differ in regions with different levels of human capital. In this study, we divided the full sample into two subsamples according to the mean of human capital, which we separately regressed. The results are shown in columns (3) and (4) in Table 10. According to the regression results, we found that digital village construction had a significant inhibiting effect on agricultural carbon emissions in areas with higher levels of rural human capital, and an insignificant effect on areas with lower levels of human capital. This result proves that the secondary digital divide, represented by digital capability, affects the dividend effect of digital village construction on green agricultural development. In the future, digital training of rural residents should be further strengthened to improve peasants’ digital application capability and break the boundary between digital village construction and rural development.

## 5. Conclusions

As digital technology matures, the Internet, big data, and other technological tools are expected to produce a comprehensive change in agricultural and rural development models. Promoting the green transformation of agricultural production and reducing agricultural carbon emissions are required to achieve the goal of carbon neutrality and mitigate the effects of climate change. In the construction of digital villages, rural digital infrastructure is improved, and digital applications are promoted to improve agricultural and rural development effectiveness and optimise the development model. From this perspective, digital construction in rural areas is an effective path to promote carbon reduction in agriculture. Through theoretical analysis and empirical testing, we found the following: Firstly, a significant negative relationship exists between the level of digital village construction and agricultural carbon emissions, and digital village construction is conducive to a reduction in agricultural carbon emissions. Secondly, the results of the heterogeneity test shows that the construction of digital villages has a stronger inhibiting effect on agricultural carbon emissions in the major grain-producing areas than in non-major grain-producing areas. The level of rural human capital is the limiting condition enabling digital village construction to influence the green development of agriculture. With a higher level of human capital, digital village construction had a significant inhibiting effect on agricultural carbon emissions.

Based on the above conclusions, further efforts should be devoted to promoting digital village construction in the future to take full advantage of the empowering effect of digital technology on the development of agriculture, rural areas and peasants. On the one hand, all types of subjects can form a joint effort to promote the construction of digital villages. Firstly, the central government needs to strengthen the top-level design of digital village construction. The current policy system for digital village construction in China is still inadequate; when given the availability of new technologies and new business models, the policy lag is particularly noticeable. For example, Ali Group established its first Taobao Village in 2009, but it was not until 2015 that the government introduced policies related to rural e-commerce. Policies related to the construction of digital villages were not announced until 2019. In the future, policy formulation work on digital village construction should be further strengthened, and the central government should play a coordinating and controlling role. Secondly, local governments should take the initiative to develop differentiated action programs based on top-level deployment. Local governments should promote the digital transformation in rural areas by improving rural digital infrastructure and promoting digital applications. A construction plan should be designed that is in line with the actual local development according to its own advantages and endowment conditions, such as creating a local agricultural e-commerce platform or promoting the upgrading of information technology in the production chain of special agricultural products, thereby forming an effective agricultural development model. Thirdly, the enthusiasm of multiple entities to participate in digital village construction should be encouraged, as well as synergy and cooperation among multiple entities. The digital transformation in rural areas will require certain financial support. Although governments at all levels invest limited financial resources, they should invest more funds in digital village construction through cooperation with social capital sources. Additionally, digital technology is the key to digital village construction, and enterprises and research institutes are important aspects of innovation in China. In 2020, 69.19% of China’s domestic valid invention patents were developed by enterprises, and 26.67% were developed by research institutions (Data source: China Statistical Yearbook of Science and Technology). Therefore, the incentive to innovate should be increased by means of liberalising the entry criteria for high-tech enterprises and providing policy support. On the other hand, digital skill training for peasants should be strengthened. The level of human capital in rural areas is relatively low, and therefore the digital capabilities of peasants are relatively poor. In 2020, the human capital index in rural areas of China was 267.82, while the human capital index in urban areas reached 2724.17 in the same period (Data source: China Center for Human Capital and Labor Market Research, Central University of Finance and Economics, China Human Capital Report 2022). The secondary digital divide, represented by digital capabilities, has become a key constraint on the effectiveness of digital technology empowerment. Therefore, increasing the level of human capital in rural areas, through targeted digital training and the strengthening of peasants’ digital literacy and digital application skills, is one of the priorities for promoting digital village construction in the future.

Of course, there are some limitations to this research. On the one hand, this paper focuses on the impact of digital village construction on agricultural carbon emissions in China, using data from mainland China as a sample. Therefore, the conclusions of this paper need to take into account the local agricultural and rural development characteristics when they are replicated in other countries. On the other hand, the lack of statistical data increases the difficulty of selecting control variables. For example, the regional innovation level (*RIL*) may be more accurate when using the number of agriculture-related patents, but the relevant data are not available. Considering that the total number of regional patents granted is closely related to the number of agriculture-related patents, the control variable regional innovation level (*RIL*) was still retained in this paper.

## Figures and Tables

**Table 1:** Agricultural carbon emission sources, coefficients, and references.

Source	Coefficient	Reference
Chemical fertiliser	0.8956 kg/kg	Oak Ridge National Laboratory, USA West and Marland [24]
Pesticide	4.9341 kg/kg	Oak Ridge National Laboratory, USA
Agricultural film	5.1800 kg/kg	Institute of Agricultural Resources and Ecological Environment, Nanjing Agricultural University
Diesel	0.5927 kg/kg	IPCC The Intergovernmental Panel on Climate Change
Irrigation	19.8575 kg/hm^2^	Wu et al. [25]
Tillage	312.6 kg/km^2^	School of Biology and Technology, China Agricultural University

**Table 2:** Digital village construction level evaluation index system.

First Level Dimension	Indicator	Specific Definition	Data Source
Digital infrastructure	Internet accessibility	Internet access per capita in rural areas	National Statistical Office
	Fixed digital device accessibility	Rural computer ownership per 100 households	National Statistical Office
	Mobile digital device accessibility	Rural mobile phone ownership per 100 households	National Statistical Office
Industry digitalisation	Digitisation of agricultural production	Ratio of number of national modern agricultural demonstration zones and industrial parks to number of county-level administrative areas	Ministry of Agriculture and Rural Affairs website, National Bureau of Statistics
	Digitalisation of sales	Taobao Villages as a proportion of administrative villages	2020 China Taobao Village Research Report
	Digitisation of the financial industry	Digital finance digitisation index	Peking University Digital Inclusive Finance Index (2011–2020)
Digital services	Digital financial services	Digital financial coverage breadth index	Peking University Digital Inclusive Finance Index (2011–2020)
	E-commerce services	Rural weekly deliveries	China Tertiary Industry Statistical Yearbook
	Mobile payment level	Mobile payment index	Peking University Digital Inclusive Finance Index (2011–2020)

**Table 3:** KMO and Bartlett’s sphericity tests.

Kaiser-Meyer-Olkin Measure of Sampling Adequacy		0.833
Bartlett’s sphericity test	Approx. chi-square	2389
	df	36
	Sig.	0.000

Notes: df means the degree of freedom, Sig means significance. Data source: calculated according to SPSS software (IBM Corp., Armonk, NY, USA).

**Table 4:** Total variance explained.

Component	Extraction Sums of Squared Loadings			Rotation Sums of Squared Loadings		
	Total	% of Variance	Cumulative %	Total	% of Variance	Cumulative %
1	5.003	55.587	55.587	3.890	43.219	43.219
2	1.618	17.979	73.566	2.731	30.347	73.566

Data source: calculated according to SPSS software (IBM Corp., Armonk, NY, USA).

**Table 5:** Variable definitions and descriptive statistics.

Type	Variable	Definition	Average Value	Standard Deviation
Explained variable	Agricultural carbon emissions	See above for detailed calculations (million tonnes)	28.809	19.686
Explanatory variable	Digital village construction level	See above for detailed calculations	4.724	2.743
Control variables	Rural economic level	Logarithmic value of per capita net income of peasants (original unit: CNY/person)	9.332	0.381
	Agroindustrial structure	Ratio of total agricultural output to total agricultural, forestry, animal husbandry and fishery output (%)	52.731	8.889
	Rural human capital	See above for detailed calculation (in years)	7.643	0.818
	Regional innovation levels	Logarithm of patents granted per 10,000 population	2.014	0.894
	Urbanisation rate	Ratio of urban population to total population (%)	56.374	12.305

**Table 6:** Impact of digital village construction on agricultural carbon emissions.

	Explained Variable: Agricultural Carbon Emissions			
	(1)	(2)	(3)	(4)
Digital village construction level	−0.395 *** (−6.584)	−0.472 *** (−4.360)	−0.259 ** (−2.544)	−0.291 *** (−2763)
Rural economic level		7.979 *** (4.445)		12.711 ** (2.442)
Agroindustrial structure		0.048 (0.977)		−0.012 (−0.256)
Rural human capital		0.185 (0.213)		−1.583 * (−1.865)
Regional innovation levels		2.661 *** (3.463)		−0.858 (−1.016)
Urbanisation rate		−0.262 *** (−3.252)		−0.165 ** (−2.121)
Constant term	30.675 *** (8.320)	−27.232 ** (−2.007)	28.936 *** (7.828)	−85.328 ** (−1.966)
Observations	300	300	300	300
Annual	No	No	Yes	Yes
Model significance test	Wald = 43.35	Wald = 76.78	Wald = 137.07	Wald = 152.74
R^2^ (within)	0.139	0.229	0.345	0.378

Note: Values in brackets are Z values. ***, ** and *, indicate significance at the 1%, 5%, and 10% confidence levels, respectively. R^2^ is the coefficient of determination that measures goodness of fit (the same applies below). Wald means Wald statistic, which is the statistic of model significance test (the same applies below).

**Table 7:** Impact of digital village construction on carbon emissions from four aspects of agriculture.

	Fertiliser	Pesticide	Agricultural Films	Fuel
	(1)	(2)	(3)	(4)
Digital village construction level	−0.193 *** (−3.291)	−0.071 *** (−4.586)	−0.010 (−0.390)	−0.005 (−0.135)
Constant term	−12.018 (−0.489)	−15.221 ** (−2.496)	−30.414 *** (−3.030)	−36.078 *** (−2.731)
Observations	300	300	300	300
Other control variables	Yes	Yes	Yes	Yes
Annual	Yes	Yes	Yes	Yes
Model significance test	Wald = 152.46	Wald = 340.58	Wald = 56.52	Wald = 41.13
R^2^ (within)	0.379	0.573	0.195	0.124

Note: Values in brackets are Z values. *** and **, indicate significance at the 1%, 5%, and 10% confidence levels, respectively.

**Table 8:** Robustness test results.

	Explained Variable: Agricultural Carbon Emissions		
	(1)	(2)	(3)
Digital village construction level		−0.287 *** (−2.738)	−0.431 *** (−3.325)
Digital village construction level 2	−6.045 ** (−2.592)		
Constant term	−85.567 ** (−1.969)	−86.643 * (−1.905)	−86.968 * (−1.902)
Observations	300	300	270
Other control variables	Yes	Yes	Yes
Annual	Yes	Yes	Yes
Model significance test	Wald = 151.47	F = 10.33	Wald = 192.49
R^2^ (within)	0.375	0.378	0.461

Note: Values in brackets are Z values. ***, ** and *, indicate significance at the 1%, 5%, and 10% confidence levels, respectively. F means F statistic, which is statistic of model significance test (the same applies below).

**Table 9:** Regression results of the instrumental variables method.

	GMM		2SLS+RE	
	Digital Village Construction Level	Agricultural Carbon Emissions	Digital Village Construction Level	Agricultural Carbon Emissions
	First Stage	Second Stage	First Stage	Second Stage
	(1)	(2)	(3)	(4)
Digital village construction level		−0.843 *** (−2.791)		−0.837 *** (−2.833)
Instrumental variable 1	27.111 *** (3.640)		26.821 *** (4.507)	
Instrumental variable 2	0.666 *** (3.783)		0.666 *** (3.981)	
Constant term				−72.518 (−1.402)
Observations	300	300	300	300
Other control variables	Yes	Yes	Yes	Yes
Annual	Yes	Yes	Yes	Yes
Model significance test		F = 9.34	Wald = 1005	Wald = 155.84
R^2^ (within)				0.331
Underidentification test	LM statistic = 34.066			
Weak identification test	Cragg-Donald Wald F statistic = 18.337			
Overidentification test	Hansen J = 0.130 (Prob = 0.719)			

Note: Values in brackets are Z values. ***, indicate significance at the 1%, 5%, and 10% confidence levels, respectively. GMM means Generalized Method of Moments. 2SLS+RE means using Two Stage Least Square for random effects model. Stock-Yogo weak ID test critical values: 10% maximal IV size 19.93; 15% maximal IV size 11.59; 20% maximal IV size 8.75; 25% maximal IV size 7.25.

**Table 10:** Heterogeneity test results.

	Explained Variable: Agricultural Carbon Emissions			
	Major Grain-Producing Areas	Non-Major Grain-Producing Areas	High Level of Human Capital	Low Level of Human Capital
	(1)	(2)	(3)	(4)
Digital village construction level	−0.731 *** (−2.654)	−0.266 *** (−2.394)	−0.290 ** (−2.039)	−0.132 (−0.416)
Constant term	−10.718 (−0.104)	−2.325 (−0.058)	14.068 (0.174)	−111.233 ** (−2.292)
Observations	130	170	186	114
Other control variables	Yes	Yes	Yes	Yes
Annual	Yes	Yes	Yes	Yes
Model significance test	Wald = 131.56	Wald = 61.56	Wald = 103.30	Wald = 81.98
R^2^ (within)	0.567	0.323	0.415	0.594

Note: Values in brackets are Z values. *** and **, indicate significance at the 1%, 5%, and 10% confidence levels, respectively.

## Data Availability

The data presented in this study are available on request from the corresponding author. The data are not publicly available due to privacy.
